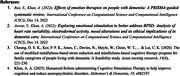# RoboZen: Humanoid Robots Administer Mindfullness Based Stress Releif Therapy to people with dementia and help them relax and improve mood and cognition

**DOI:** 10.1002/alz.094322

**Published:** 2025-01-09

**Authors:** Arshia A Khan

**Affiliations:** ^1^ University of Minnesota Duluth, Duluth, MN USA

## Abstract

**Background:**

In this study humanoid robots were programmed to deliver mindfulness‐based stress relief therapy to individuals with dementia. Precisely programmed, these robots aim to enhance well‐being, providing tailored interventions for relaxation, mood enhancement, and heightened cognition in dementia care, showcasing technology’s promise in improving overall quality of life [1, 2, 3, 4].

**Methods:**

In this study, the humanoid robot was programmed with a carefully AI based design protocol incorporating calming activities, guided breathing exercises, and interactive engagement, tailored to the cognitive abilities and preferences of individuals with dementia, to effectively deliver mindfulness‐based stress relief therapy. This mixed‐methods study assessed the effectiveness of humanoid robots in delivering mindfulness‐based stress relief therapy to individuals with dementia. The sample included 9 participants diagnosed with dementia residing in nursing homes. Ethical approval was obtained from the Institutional Review Board. The intervention involved a humanoid robot delivering mindfulness‐based stress relief sessions. Quantitative data, obtained through pre‐ and post‐intervention assessments, included standardized scales measuring stress levels, mood, and cognitive function. Physiological indicators such as heart rate variability and electrodermal activity were recorded using wearable sensors during sessions. Qualitative data were gathered through participant observations and post‐session interviews to capture subjective experiences and perceptions of humanoid robot‐administered therapy. Thematic analysis identified recurring patterns and insights. The study aimed to offer insights into the feasibility, acceptability, and potential benefits of integrating humanoid robots into dementia care for stress relief, contributing to innovative approaches in dementia interventions.

**Results:**

Analysis of data from participants undergoing humanoid robot‐administered mindfulness‐based stress relief therapy showed significant improvements. The Brief Introspection Mood Scale (BMIS) indicated a highly significant enhancement in mood scores post‐intervention. Participants reported consistently positive shifts in emotional states, supported by electrodermal data showing reduced stress levels during and after sessions. Cognitive assessments, using the Montreal Cognitive Assessment (MoCA), consistently demonstrated improved cognitive function following the intervention.

**Conclusion:**

In conclusion, this study demonstrates the promising efficacy of humanoid robot‐administered mindfulness‐based stress relief therapy, revealing significant improvements in mood, physiological stress indicators, and cognitive function among individuals with dementia. This offers a valuable avenue for holistic care interventions.